# Hospital and Institutional News

**Published:** 1915-07-31

**Authors:** 


### HOSPITAL AND INSTITUTIONAL NEWS.

### A SANATORIUM FOR MUNITION WORKERS.

AROUND the proposal to use as a sanatorium for sailors and soldiers suffering from tuberculosis a large house situated in a public park, there arose an animated discussion at a recent meeting of the Newport Town Council. A deputation was received from the Welsh National Memorial Association, and it was urged that the Council should grant the use of Beechwood House for the purpose of treating tuberculous sailors and soldiers. Sir Garrod Thomas and Dr. Marcus Patterson were among the members of the deputation, which was composed of prominent officials of the Association and the Insurance Committee. The major part of the discussion centred around two points. First, the suitability of a building in a public park, and the degree of risk to the public from tuberculous patients residing in such a place. Secondly, the question whether the accommodation should be restricted to sailors and soldiers. The first-mentioned point was eventually disposed of satisfactorily, but there was a great deal said on the second subject of discussion. Finally, an amendment was agreed to which allowed the fifty beds available to be reserved exclusively for the use of sailors, soldiers, and munition workers. It was felt that after a comparatively short time there would be, unfortunately, no difficulty in finding patients of this restricted class to keep the whole of the beds fully occupied. A considerable amount of local opposition appears to have existed against the proposal to use the house in the park, even though it was to be occupied by tuberculous "war workers," for the duration of the war only, and it is not a little strange to find that the matter of risk to the public should require to be reassuringly negatived for the benefit of some of the members of so important a town council. Some people are slow to realise that a consumptive sheltered and cared for is less dangerous than the consumptive at large.

### MOTHERS' RIGHTS AND CHILDREN'S NEEDS.

THE Lambeth Board of Guardians have now in working order a scheme which has entailed much thought, time, and expense. For many years the Local Government Board has been urging them to remove from their workhouses the infants and young children, and they have converted a part of their buildings close to their children's school at Norwood into a nursery and clinic for the very young. Considerable opposition was met with at the outset in removing the children such a long distance away from their mothers, but arrangements have been made enabling the latter frequently to visit their babies. The salubrious air of Norwood is preferable to that of the closely confined district in which the workhouses are situated, and their health should be materially benefited by the change. The medical arrangements are under the control of Dr. A. N. V. Johnson, a lady who for many years has been the medical officer at the school, and who has evinced the greatest interest in the welfare of the young people committed to her care. But it is essential for the success of the scheme that arrangements should be made so that the mothers do not feel deprived of their children.

### WEEK-ENDS FOR INFIRMARY SUPERINTENDENTS.

THE difficulty of obtaining assistant medical officers for Poor-Law infirmaries is again exemplified in the case of the Southwark Board of Guardians, although the Local Government Board has been induced to increase the salary attached to the office. The Board has been advertising for some weeks, but has received no replies, and, in consequence, additional labours have been thrown upon Dr. H. W. Bruce, the medical superintendent at the infirmary. There are no permanent assistants on his staff, and he has been forced to relinquish the remainder of his annual leave. The Board has granted him permission to have week-ends off duty, as it might be found impossible to arrange for the administration of the infirmary should he be away for a lengthy period. It is evident that the war may seriously affect those who have loyally stood by their authorities in order to see that the sick poor of the Metropolis are not neglected, and it is only fair to them and the manifold extra duties they have been called upon to perform that their services to the community should be fully recognised now by such holidays as week-ends can conveniently secure, and when the war is at an end by an adequate public acknowledgment.

### MEDICAL CERTIFICATES AND OLD-AGE PENSIONS.

A MATTER which is a source of difficulty to many Poor-Law medical officers has recently been under discussion by the Bromsgrove Guardians. If an old-age pensioner is admitted to a Poor-Law institution his pension is stopped unless his admission and retention in the institution are due primarily to his need of medical or surgical assistance. It is customary for the pension officer to send to the medical officer of the institution a form which includes the following three questions: (1) What is the nature of the illness from which he is suffering? (2) Is his maintenance in the institution due primarily to his need of medical or surgical assistance? or (3) Is it due primarily to his insufficient means and to his need of care and attention which he is unable to obtain outside, such medical and surgical relief as he requires being incidental thereto? It is in answering these last two questions that the medical officer often finds himself in difficulty. In many cases, of course, there can be no difficulty. Where the pensioner is acutely ill or where he is receiving no medical or surgical attention the answers are obvious, but there remain a large group of cases where the patient is not seriously ill, but requires some form of treatment. Questions 2 and 3 can then be answered only by someone cognisant of the home conditions of the patient—knowledge which is not usually in the possession of the medical officer. Dr. Rowlands, the workhouse medical officer at Bromsgrove, solved the difficulty by declining to answer the questions at all, and the matter was referred to the Local Government Board by the Chairman of the Local Pensions Committee.

### "I DO NOT KNOW."

The Board admits the existence of the difficulty in many cases, but points out that in some cases Dr. Rowlands had certified the nature of the illness to be old age, and suggests that in such cases it would be justifiable to infer from the absence of the mention of any specific illness that the pensioners were maintained in the infirmary for care and attention rather than for actual medical or surgical assistance, and hopes that this expression of their view will assist in removing Dr. Rowlands' difficulty. We fear that this is hardly likely to be the case. Old age may be an indefinite diagnosis, but the condition is one which often requires medical advice and observation, as well as ordinary care and attention. The medical officer is asked to say which of these two requirements has been the predominating factor in leading the pensioner to seek institutional relief. In the absence of full knowledge of the circumstances of the case it is impossible to decide with certainty. The only answer the medical officer can conscientiously give is "I do not know." And this simple answer has the additional advantage that no one can dispute it, and the onus of deciding the pensioner's right to retain his pension is then placed where it should rightly rest—upon the pension officer.

### THE DISPENSING OF MEDICINE TO THE PUBLIC.

On several occasions and in divers places complaints have been made that the facilities afforded to insured persons for obtaining medicines prescribed by panel doctors, are insufficient by reason of the fact that panel chemists close their shops too soon on certain days of the week. The latest complaint comes from Glasgow, where a lengthy discussion on this very subject has been occupying space in the local Press. The indictment was made by the "hon. sec." of the Scottish Branch of the Panel Medico-Political Union, who, in a letter to the *Glasgow Herald*, alleged that the West of Scotland Chemists' Association by their application to the local authority to close chemists' shops at eight o'clock on certain days of the week had imperilled the pharmaceutical service under the Insurance Act, since "no persons could have medicines or dressings after eight o'clock at night, until nine o'clock the following morning." This letter drew replies from a number of panel chemists, and the correspondence which began at the end of June was prolific. To a large extent the whole business is based upon a misunderstanding of the Shops Act. Under that Act the local authority has power to order the closing of shops at certain hours, but has no power to prevent the selling of medicines and surgical appliances after the hour when the shop is closed for the sale of the many "side lines" which are part and parcel of the modern chemist's trade.

### A GRAVE CHARGE.

Unless Glasgow is altogether different from other places in Great Britain, there will be some person on duty at most of the chemists' shops until a very late hour, while at many of them medicines will be available at all hours of the day and night. But if, as is alleged, the facilities afforded to insured persons are insufficient, steps should be taken to induce the Insurance Committee to take the necessary action to ensure that the ground for the complaint is removed. The insured person is furnished by the Insurance Act with a remedy for grievances of this kind, and it would perhaps have been preferable to have made the appeal to the properly constituted body, rather than to the public through the lay Press. Incidentally it is hinted by the honorary secretary of the Scottish Branch of the Union that there is a suspicion among insured persons that the chemist does not dispense what the doctor prescribes. This is a very grave charge to make, and if there is any ground for it, it should be made to the proper authorities. If, on the other hand, the charge is merely based upon the tittle-tattle of scandal-mongering patients, it is unworthy of any member of the medical profession to write a letter to the Press about it.

### FINE FOR AN UNQUALIFIED "DOCTOR."

The Medical Defence Union has succeeded in bringing to book an unqualified "surgeon," who was fined £30, and ordered to serve, in default of payment, one month's imprisonment. The man, it appeared, had signed two "doctor's" certificates on behalf of a soldier who pleaded illness when charged with failing to support his wife. The defendant described himself as M.B. of New York and M.D. (London), as a surgeon and a resident dental surgeon. He was not in the current Medical or Dental Registers, and in 1906 was stated to have been fined £5 for forging a death certificate. In giving evidence, a constable declared that he had known the defendant as a "doctor" for fifteen years. The defence was that the defendant was a registered dental surgeon as far back as 1878, but had not troubled to place, or keep, himself upon the register. He had been a doctor's assistant several times, but had never pretended to be a medical practitioner. He attended the soldier as an ordinary man without any medical knowledge. We have previously drawn attention to the carelessness with which certain medical men accept on trust the qualifications put forward by applicants for assistantships, or even partnerships. The present case seems to show that the accused in this instance also had in the past traded on similar negligence on the part of those to whom he referred.

### THE INSURANCE DEADLOCK IN BELFAST.

In the tuberculosis scheme for the City of Belfast a serious hitch appears to have arisen between the Corporation and the Insurance Committee. This latter body should, apparently, have handed over to the Corporation a sum of about £10,000 a year, in return for which the City Fathers were to undertake to treat every tuberculous person in Belfast, whether insured or uninsured. At least one hundred insured persons have already been admitted into sanatoria, for whose treatment the Corporation have not received a single penny; and the Insurance Committee have been notified that, unless payment were forthcoming, treatment to insured persons would be refused. It was stated that both bodies were paying £600 per annum to their respective tuberculosis officers—an apparent waste of £600 a year. Some attempt at an agreement had been made a long time ago, and the Insurance Committee had consented to leave the matters at issue to the Insurance Commissioners, while the Corporation agreed to leave their side of the question to the Local Government Board. But these bodies seem to have been disputing ever since, and to have introduced even secondary causes for dissension. The result is that neither the insured nor the uninsured tuberculous persons are being adequately treated in Belfast, either in sanatoria or in dispensaries, and the Corporation are losing the use of a sum of £10,000 a year for these purposes. One of the main points at issue relates to the demand by the Belfast Insurance Committee for representation on the Hospitals Committee of the City Council. This committee, which, to a large extent, controls the tuberculosis scheme, is prepared to give way in this matter, but some objection has been raised by the Local Government Board. Such a state of affairs is highly discreditable to the bodies involved in it, and the higher the authority the greater the blame for not finding a way out of the *impasse*.

### THE ROYAL ARMY MEDICAL CORPS AND PROMOTION.

The House of Commons lately has produced an exceptionally interesting crop of questions, of which those affecting hospital and allied matters have already been referred to in our columns. The following is also of interest. In reply to Sir Clement Kinloch-Cooke, Mr. Forster says that it has been decided to promote to the rank of captain all lieutenants of the Special Reserve and Territorial Force, Royal Army Medical Corps, who have given six months' mobilised service, with effect from April 1 last, and steps are now being taken to carry out this decision.

### THE ECONOMICAL WEAPON AGAINST TUBERCULOSIS.

Various suggestions have been made by Insurance Committees as to the best method of cutting down expenditure in connection with the treatment of tuberculous persons. Restrictions have been proposed in three principal directions—namely, less institutional accommodation, no treatment for dependants, and the cutting off of eggs and milk from domiciliary cases. It is unfortunate that so many persons lose sight of the tuberculosis problem when dealing with individual cases. The one thing which must not be restricted is the accommodation for the advanced case. In fact, reliable authorities maintain that the cheapest, most direct, and most efficient weapon in the war against tuberculosis is the segregation of advanced cases. No inducement, therefore, should be given to any such patient to remain at home, if there is a bed available in some institution. Unfortunately there are not yet enough beds for such cases, and many must stay at home. Then it should be arranged that no supplies are given to any patient who refuses to do his utmost to reduce the risk of infection to his family. Such an one ought not to be assisted to increase the danger of his life to the health of others. So, if he wants milk and eggs he must go where he may be given them under proper supervision. Any community which has provided no beds for segregating advanced cases is merely sentimentalising and finicking with the tuberculosis problem.

### A HARD-WORKED MENTAL STAFF.

In view of the work which the putting into operation of the Mental Deficiency Act has involved upon the staff of the Mental Hospitals Committee of the London County Council, that body has decided to make a grant of £500 to the staff, of which £300 is to be given to Mr. H. F. Keene, the clerk of the committee. It is pointed out that when the Act came into operation the first necessity was to determine the lines upon which it was to be administered, and numerous difficulties presented themselves, which involved fundamental questions of principle. It was vital to the successful working of the Act in the future that these questions at the outset should be determined carefully, and that the basic principles of the Act should be established thoroughly and upon broad lines. The work, therefore, was such that practically none of it could be delegated. It comprised no routine work, for routine had to be established. In addition, their ordinary duties under the Lunacy Acts were not lessened and could not be neglected. Later, when a system was beginning to be evolved, war broke out and members of the staff enlisted. Thereupon, in view of the difficulty which was found in getting efficient clerks who were non-enlistable, the idea of engaging an additional permanent staff for mental deficiency work had to be abandoned.

### THE PROBLEM OF RESIDENCE.

An example of the difficulties which have to be solved by the Mental Deficiency Committee is that attending the precise meaning of the words "place of residence." There are in rescue homes and similar institutions in London numbers of persons, some of whom are defective, whose only association with London is the fact that they have been sent there for care. The same thing is true, *vice versa*, of such institutions in other districts where London defectives are being maintained. The need for a common ground for agreement as to what constitutes residence is obviously urgent, as the Council may be faced with difficulty in dealing with another authority whose view as to the meaning of residence does not coincide with that held by the Council. In such a case the Council might be expected to accept responsibility not only for those cases in which it is prepared to take action (which perhaps the other authority would be willing to deal with), but also for those which, in the Council's opinion, it is the duty of the other authority to provide. The Board of Control is therefore to be urged to define the meaning of "residence," and unless an arrangement be arrived at within twelve months the County Council intimate that they will decline to accept responsibility for defectives who in their opinion ought to be a charge upon another local authority.

### A DOCTOR AS GRAVE-DIGGER.

During a recent outbreak of typhus fever in Donegal, it is reported that something of a panic overtook the inhabitants of the affected neighbourhood. In one case the friends refused to return to the house in which a body of a victim to the disease was lying, and nobody could be found to undertake the burial. In the end this office had apparently to be performed by the medical officer, Dr. C. E. R. Gardiner, who was assisted by two courageous nurses. A grave was dug in a field by these three persons, and they themselves interred the body.

### THE CLOSING OF SCHOOLS OWING TO INFECTIOUS DISEASE.

At a recent meeting of the Cornwall Sanitary Committee the Medical Officer of Health reported that he had ordered the closing of many infants' departments on account of the prevalence of measles. Several of the members protested against this course being adopted, and pointed out that the children met in the streets, in chapels and Sunday schools, and places of entertainment where the possibilities of infection were as great, if not greater than at school. The Medical Officer justified his action on the grounds that so many children were affected that the school work was disorganised. Speaking generally, the closure of schools in the presence of an outbreak of infectious disease is to be deprecated. The School Memorandum of 1890 points out that, if the attendance at a school be greatly reduced by the absence or exclusion of a number of children owing to infectious disease, it may be found better to close it altogether. A second material consideration is the amount of opportunity for intercommunication between the members of different households elsewhere than at school. In sparsely-populated rural districts the closing of the school is likely to be effectual, but it is less likely to be useful in a town or compact village. The influence of school attendance in the case of scarlet fever and diphtheria was shown by Sir Shirley Murphy in 1894 and 1898, when he demonstrated the existence of a marked depression in the curves of incidence of these diseases in children of school age synchronising with the August holidays. On the other hand, the school may be made a means of detecting early cases of infectious disease, and so aid in limiting the spread of infection. The clinical thermometer will enable the teacher to pick out cases which should be under medical observation, and thus lead to the segregation of infected children earlier than might occur if the child were at home or running about the streets. If at the same time the school work were conducted as far as possible under open-air conditions, the risk of infection would be minimised and the general health of the children would benefit.

### WALTHAMSTOW HOSPITAL.

The Walthamstow Hospital has recently completed a much-needed addition to its equipment in the shape of an *x*-ray room, which has been built and is now fitted with modern apparatus, couch, dark-room, annexe, etc., as the result of the proceeds of a Garden Fête. This was held at "Highams," Woodford, and was so successful that it provided not only the above, but also a balance sufficient to pay a considerable debt.

### THE LOCAL GOVERNMENT BOARD AND CONTRACTS.

There has been a general desire on the part of public authorities to treat their contractors generously, in view of the abnormal conditions that have prevailed, and in order to provide supplies for their large institutions. The policy has, so far, worked with success, for public authorities are not always prone to deal leniently with their contractors, and the concessions and extra amounts that have been granted have done much to create and foster a spirit of tolerance that hitherto has often been lacking. A letter addressed to the Camberwell Borough Council, however, may cause public authorities to pause ere they grant extra sums to their contractors for contingencies due to the war. The Council asked permission to pay their contractors extras, and a reply has been received from the Local Government Board stating that, in the exceptional circumstances, should the Council decide to make any payment to a contractor in excess of the amount due under the contract, it should be considered by the Public Auditor at the audit as to its reasonableness, as in the case of other payments for goods supplied or for work done. The Board did not think that payments of this kind should be sanctioned by them before the audit, so as to withdraw them from the auditor's review. The direct meaning of the latter is that should the auditor in his wisdom think that an authority had paid a contractor too much in the way of extras due to the war, he will surcharge the authority with the amount. It seems evident that such a far-reaching decision would prevent the authorities from granting any extra remuneration. Indeed, many authorities will seriously consider their position ere they become responsible for sums with which the Public Auditor may surcharge them.

### THE CHRONIC INSURED PATIENT.

On the whole, it may be said that complaints on behalf of insured patients against the behaviour of panel doctors are infrequent, and the view that Carnarvonshire Insurance Committee lately expressed on a case submitted to them shows how the relation of the two is regarded in Wales. One complaint was submitted in which it was alleged that a panel doctor had spoken disrespectfully to the wife of an insured patient, but as the doctor was stated to have apologised the matter dropped. Another complaint inferred negligence on the part of a panel doctor. It had therefore to be determined whether the alleged negligence had occurred, and, if so, whether it had been such as to cause the patient the expense of calling in another medical man. The Committee found that, on the evidence before it, the patient's illness had not been aggravated by negligence, but, while recognising that the doctor was hard pressed at the time, they expressed the opinion that it would have been more satisfactory if the doctor had seen the patient between two specified dates. Further discussion revealed the fact that the patient was suffering from a chronic complaint, and the Sub-Committee had come to the conclusion that even if more visits had been paid the doctor could have done no more. The Chairman remarked that it was the first real complaint brought before them in the course of their three years' work. It was essential that every insured person should have " not only every necessary treatment, but also every respectful treatment." On the other hand, no undue advantage should be taken of the time or service of panel practitioners. These two desiderata are not easy to obtain where a chronic case is in question, as private practice as well as panel practice sufficiently shows. Harmony can be preserved only by the display of tact both by the patient and his medical adviser. The only thing that can be said is that, under contract practice, the panel practitioner has not the individual stimulus which is his where a private case is concerned. On the other hand, a chronic case, under contract practice, is more tempted to make unreasonable demands on the time of his doctor than he would were he consulting a doctor as a private patient.

### A NEW DEPARTMENTAL COMMITTEE ON POOR-LAW ORDERS.

A Departmental Committee is now sitting to consider the provisions of the Poor-Law Orders relating to the appointments, qualifications, tenure of office, duties, and remuneration of Poor-Law officers. The inquiry will not extend to officers of institutions, such as separate infirmaries or children's homes, to which the Poor-Law Institutions Order of 1913 does not apply, but it will include other institutional officers and all non-institutional officers. The Committee has invited the Association of Poor-Law Unions and the National Poor-Law Officers' Association to give evidence. The reference to the Committee is a very wide one, and its recommendations may form the basis of far-reaching changes. Mr. T. Theodore Dodd, in a letter to the Press, points out that the President of the Local Government Board has been asked in the House of Commons, and has refused to promise, that any Poor-Law Order drafted by this Committee would be laid before Parliament before sanction. Mr. Long was also asked if he would refuse sanction to any proposed Order taking away or diminishing the responsibilities and liabilities of relieving officers for the relief of the poor, especially in regard to such as are in a condition of sudden or urgent necessity; but to this he gave no reply. Mr. Dodd draws attention to the fact that four of the members of the Committee were also members of the Committee which drew up the drafts of the Relief Order and the Poor-Law Institutions Order, both of which had to be greatly amended. He suggests that members of all parties should protest against any revision of the Poor-Law Orders during the war. We cannot agree that Poor-Law reform should thus be held up, but we do most strongly support the view that no fresh Order should be promulgated until the evidence collected by the Committee and its recommendations have been fully considered and discussed in the Press and in Parliament.

### AN ILL-FOUNDED RUMOUR ABOUT A HOSPITAL SHIP.

In times of public excitement, particularly in war, after the first phase of public enthusiasm has necessarily given way to a more searching glance at what has been actually done, rumours of mismanagement of every kind are often rife. The hysteria of enthusiasm, always an ingredient in popular movements, gives way, among certain sections of the Press and public, to the hysteria of rumour and criticism. Mr. Tennant's official denial in the House of Commons concerning a rumour affecting the hospital ship *Oxfordshire* may be given, therefore, in the words which he used when replying to a question from Mr. Watt. There was no truth, Mr. Tennant remarked, in the allegation that on the hospital ship *Oxfordshire*, from Havre to Southampton, on March 19, 60 wounded British soldiers, the majority of them from the Black Watch and 6th Gordon Regiments, were taken out of their cots to make room for 60 Germans; that one of the British soldiers subsequently died in Perth; and that, in addition, the Germans were supplied with fresh eggs and bread while the British wounded soldiers had only biscuits. He was glad of the opportunity of correcting a rumour, as unpleasant as it was ill-founded, which had obtained widespread credence.

### AWARDS OF THE MILITARY CROSS.

The King has been pleased to approve of a further list of awards for gallantry and devotion to duty in the field. The Military Cross has been awarded to the two following members of the Royal Army Medical Corps:—To Lieut. William Kelsey Fry, R.A.M.C., " for conspicuous gallantry and devotion to duty at Festubert between May 16 and 18, 1915, while carrying out his work under heavy fire; he was himself wounded while attending to others "; and to Lieut. David James Sheires Stephen, M.D., R.A.M.C., " for conspicuous gallantry and devotion to duty in attending to the wounded under heavy shell fire on several occasions, notably on the night of April 23 and on May 8, 1915; he has usually performed his gallant work single-handed, and by his cheerfulness and pluck has encouraged all around him."

### SECRETARIAL POSTS FOR "THE UNFIT."

THE difficulty of securing what are now becoming known as non-enlistable clerks is being felt by hospitals as well as by business firms. Thus St. Mary's Hospital, Paddington, has been publishing an advertisement to the effect that certain "temporary vacancies" exist in "the secretary's office owing to the departure of three clerks for the war. This is an opportunity," the announcement proceeds, "for anyone, man or woman, competent to perform office duties, but unequal to military service. No one physically capable of military service will be accepted." It will be observed that, from the terms of the advertisement, applicants who are married men, if physically fit, would appear to be excluded.

### SIR R. BORDEN AND CANADIAN WAR HOSPITALS.

THE visit paid by Sir Robert Borden to the Front included the inspection of several Canadian hospitals, the chief of which is, perhaps, that known as No. 1 Canadian General Hospital, of which the commanding officer is Colonel Murray MacLaren. Forming the centre of what has been described as a hospital town, near Etaples, the design comprises Indian tents arranged in long corridor wards, each of which can accommodate sixty-four patients. The construction and decoration of the tents, if the term may be allowed, comprises triple walls, lined with old gold, and their breadth has been remarked on as an attractive feature. Electric lighting has been installed throughout. The Canadian hospitals comprise three general hospitals in France, where there is also a clearing hospital, besides Red Cross distributing centres. The 2nd Canadian Stationary Hospital is situated at Le Touquet, and occupies not only the hotel, but also the links, on which tents have been erected. In England, moreover, three Canadian Army hospitals have been established.

### THE EXHIBITION OF ARTIFICIAL LIMBS.

THE interest of orthopædic surgeons was inevitably aroused in the international exhibition of artificial limbs held last week at Queen Mary's Auxiliary Hospital, Roehampton House. The display was remarkable from the fact that examples were shown of the work done in the countries of the Allies, in America, Sweden, and Denmark. Particulars of the institution, which was visited by Princess Louise and Queen Amelia on Tuesday last, have already appeared in our columns. The hospital is intended for members of the Forces who have lost their limbs during the war, and a conference was held on Tuesday afternoon, presided over by Sir Watson Cheyne, to discuss the question of providing such men with the most suitable surgical appliances, so as to fit them as far as possible for any work which they may be able to undertake. Orthopædic surgeons and representatives of the firms engaged in the manufacture of artificial limbs were present, and had the opportunity of listening to Sir Arthur May, Director-General of the Navy Medical Service, and Surgeon-General Russell, who attended on behalf of Sir Alfred Keogh. Sir Arthur May stated that artificial limbs were required for nearly 1,000 men now waiting for them. A committee of surgeons is reporting on the best makes of artificial limbs, and the prizes are awarded by the committee. The value of the new institution, as already pointed out in these columns, should consist in providing the disabled soldier with a means of having his artificial limb expertly fitted, and also, which is no less important, in teaching him how best to make use of it. This necessitates skilled supervision, and is a great improvement, as was pointed out in the speeches, on the old method by which a man was measured and then, often after a long interval, provided with a new limb, which was often discarded owing to the absence of proper instruction in its use. The demonstrations of the adaptability of artificial limbs under the control of those practised in their use was very suggestive. Except to those whose technical knowledge makes them acquainted with the possibilities of these limbs, the results appear astonishing. It may be pointed out that while Roehampton House can accommodate some 200 cases, accommodation for twenty-five officers is available at Dover House, Roehampton, and a further seventy-five beds are to be used in connection with the former. It is hoped that similar institutional treatment may be organised in Scotland.

### THIS WEEK'S DRUG MARKET.

IN spite of the near approach of Bank Holiday, business in the drug market remains active. Of important changes in value there have been few during the week, but the general upward tendency of values continues. More definite reports on the crops of English medicinal herbs are now available, and it appears that many of the crops have suffered from the prolonged drought in the spring. Both belladonna and henbane promised well, but, owing to lack of rain at the time when it was wanted, the crops were comparatively poor. Peppermint has not done well, and the yield of oil will be short and prices no doubt high. A good supply of lavender is expected, and chamomiles give promise of a fair crop. Among the chemicals that have advanced in price are boric acid and borax. The value of cocaine is not so firmly maintained. Sulphonal is dearer. Opium is unchanged. Citric acid and tartaric acid are likely to advance in price. Buyers of cod-liver oil are reluctant to operate at the high prices quoted. It is reported from Norway that the heavy demand from Russia and Germany continues, and that stocks of oil are low.

### CHARING CROSS HOSPITAL.

WITH reference to the article which we published last week on the subject of Women Students at Charing Cross Hospital, the statement that the Council of the Charing Cross Hospital Medical School had decided to admit women students was premature. The matter has been under consideration for some time, but no decision, at the time of writing, has actually been made. We regret that we were misinformed on this point, and are glad to take the first opportunity to state the correct position.

